# Atrial Fibrillation for the Neurologist: Preventing both Ischemic and Hemorrhagic Strokes

**DOI:** 10.1007/s11910-018-0813-y

**Published:** 2018-02-06

**Authors:** Elif Gokcal, Marco Pasi, Marc Fisher, M. Edip Gurol

**Affiliations:** 1 Department of Neurology, Bezmialem University, Istanbul, Turkey; 2 Department of Neurology, Hemorrhagic Stroke Research Program, Massachusetts General Hospital, 175 Cambridge Street, Suite 300, Boston, MA 02114 USA; 3 Department of Neurology, Beth Israel Deaconess Medical Center, Boston, MA USA

**Keywords:** Atrial fibrillation, Ischemic stroke, Intracerebral hemorrhage, Left atrial appendage closure

## Abstract

**Purpose of Review:**

This review aims to help neurologists managing atrial fibrillation (AF) patients who had an ischemic stroke and/or with intracranial hemorrhage (ICH) markers, therefore at high embolic/hemorrhagic risks.

**Recent Findings:**

Implantable loop recorders have substantially improved the accuracy of AF detection. Recent research yielded a set of powerful neuroimaging markers that can stratify ICH risk. Direct oral anticoagulants (DOAC) are easier to use with a lower ICH risk than warfarin in a general AF population. Finally, the FDA-approved left atrial appendage closure (LAAC) with the WATCHMAN device provides an option without the need for life-long anticoagulation.

**Summary:**

In this review, we introduce the concept of preventing both ischemic and hemorrhagic strokes in AF patients through accurate AF diagnosis and stratification of both embolic and ICH risks. LAAC can be considered in patients at higher hemorrhagic risks while warfarin/DOAC use should be individualized in the majority of AF patients at a low risk of bleeding.

## Introduction

**Fig. 1:**
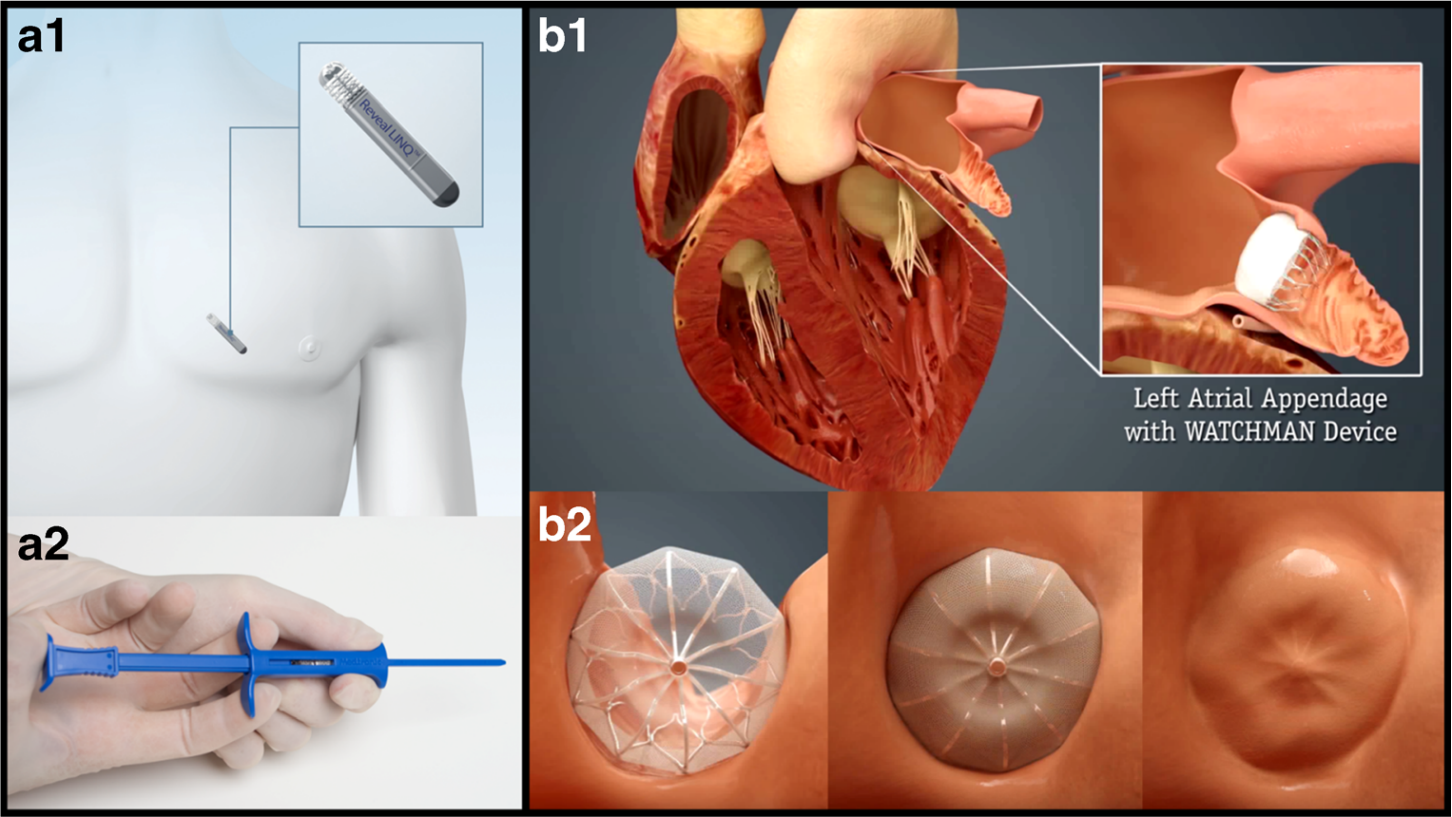
Implantable loop recorder and left atrial appendage closure devices. **a** The schematic of an implantable loop recorder placed in position (A1) and the simple delivery system used for its insertion (Reproduced with permission. Copyright ©2017, Medtronic, Inc). **b** The left atrial appendage before and after placement of the WATCHMAN left atrial appendage closure device (B1) and a schematic of the progressive covering of the device with a tissue layer (B2) typically occurring over 45 days. (Image provided courtesy of Boston Scientific. © 2017 Boston Scientific Corporation or its affiliates). All rights reserved by their respective owners

Atrial fibrillation (AF) is the most common cardiac arrhythmia with an estimated 2.7–6.1 million people in the USA having this condition [1]. Risk factors for AF include advancing age, hypertension, diabetes mellitus, smoking, obesity, heart failure, and valvular heart disease. Among Medicare Fee-for-Service beneficiaries in the USA, 2% of the people younger than 65 years of age have AF, while about 9% of the population over 65 have AF [2]. The lack of adequate screening in the general population and the much higher prevalence found in high-risk populations with advanced monitoring (Fig. 1a) suggest that the currently available incidence/prevalence findings are underestimates. When the consequences of the rapidly aging population are added to this equation, it is estimated that there will be 12.1 million AF patients in the USA by 2030 [3] and 17.9 million in Europe by 2060 [4].

Regardless of its pathogenesis, AF is a very important risk factor for stroke, independently increasing the risk about 5-fold in all age groups [5]. The mechanism of ischemic stroke in AF is embolization from intracardiac clot formation, with the left atrial appendage (Fig. 1b) being the most common site for clot formation in non-valvular AF (NVAF) [6]. The embolic stroke risk is eight times higher than systemic embolism in AF based on pooled data from four large contemporary randomized clinical trials (RCT) of anticoagulation in AF [7]. AF-related embolic strokes are typically more severe than other ischemic strokes and they are associated with a significantly higher risk of recurrence and poorer long-term outcomes [8, 9]. Therefore, both primary and secondary stroke prevention strategies are very important to decrease morbidity/mortality in patients with AF.

The last few years have seen important advances in the detection, treatment, and stroke prevention efforts in the field of AF. Newer and safer direct oral anticoagulants (DOACs) have been approved for stroke prevention in NVAF but recent data show profound underuse of preventive strategies in AF patients [10•]. Part of the problem stems from the lack of a multidisciplinary approach that should ideally involve neurologists, cardiologists, internists for many AF patients, and other specialities such as hematology and gastroenterology in selected situations. The neurologist should be an important member of the AF management team, adding valuable input as to the diagnosis of AF-related ischemic stroke, understanding both the embolic and hemorrhagic stroke risk in individual patients and selection of appropriate preventive measures. Neurologists primarily manage patients who either have had a stroke, transient ischemic attack (TIA), or oral anticoagulant-related intracranial hemorrhage (OAC-ICH), therefore a higher risk population when compared to general medical/cardiology practices. The neurologist should thus be the ideal physician who can shape shared decision-making discussions with patients to decide about the best management in the light of up-to-date scientific evidence while taking the patient’s values into the account.

**Fig. 2:**
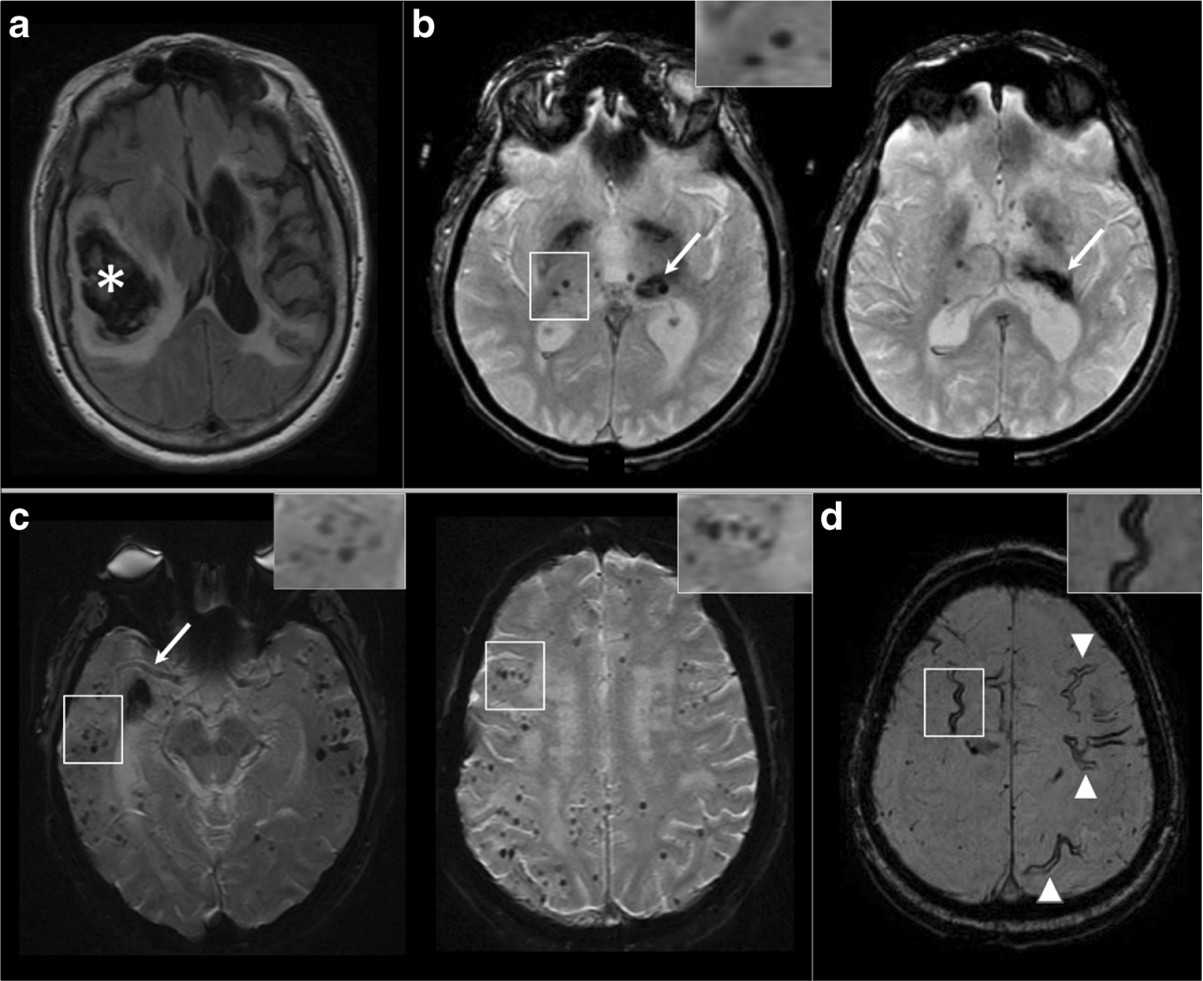
Hemorrhage-prone small vessel disease markers. **a** Axial FLAIR sequence; large right parieto-temporal hematoma with extensive edema causing midline shift (star) and also extensive periventricular white matter hyperintensities. **b** Axial T2* sequence showing a patient with a left thalamic hematoma (arrows) and bilateral deep cerebral microbleeds (inset). **c** Axial T2* sequences showing many strictly lobar microbleeds (inset) in a patient with a right temporal hematoma. According to the Boston criteria, this patient fulfills criteria for cerebral amyloid angiopathy. **d** Extensive cortical superficial siderosis (arrowheads and inset) visible in T2* sequence

This review article will focus on cutting-edge advances in AF detection and treatment, with the aim to help the neurologist contribute maximally to the management of these complicated patients. Stroke prevention in AF has been classically seen as ischemic stroke prevention despite the exceedingly high mortality/morbidity of ICHs that occur in the setting of life-long anticoagulation. One of the major advances in the realm of stroke neurology has been a better understanding of etiologies of ICH and stratification of both first time and recurrent ICH risk based on imaging and other data (Fig. 2). A recently approved left atrial appendage closure (LAAC) procedure that uses the WATCHMAN device can obviate the need for life-long anticoagulation while giving embolic protection to the AF patients. In this review, we introduce the concept of all stroke (both ischemic and hemorrhagic) prevention in AF. Diagnosing or ruling out AF accurately is important to make sure that patients receive appropriate preventive measures, so advances in detection will be presented. We will then discuss stratification of not only embolic risk but also ICH risk and the modern approaches to prevention in this framework. The current review article will mainly focus on NVAF, but brief updates on valvular AF and AF with other concomitant pathologies will be discussed at the end of this text.

## Advances in the Detection of AF

Appropriate diagnosis of AF is key to optimal management. Treating patients who have AF with appropriate stroke prevention measures and not using anticoagulants or LAAC in patients who do not have AF maximize the odds of preventing both ischemic and hemorrhagic strokes, the main theme of this review. Individuals at high risk of AF include patients who had a stroke or TIA that is more likely to be of cardioembolic origin based on clinical and radiologic assessment as well as patients with rhythm-related symptoms who have risk factors, family history, or other imaging markers of AF (such as left atrial dilatation). Overall, we highly encourage using the most sensitive and long-term rhythm monitoring approaches, specifically the implantable loop monitors in these patients if electrocardiography (ECG) and inpatient telemetry or a Holter monitor were negative.

For years, AF has been diagnosed using an ECG in patients found to have irregularly irregular heart rhythm or during screening. Historically, the first report of irregular pulses detected with the aid of stethoscope was in 1827. The main diagnostic breakthrough was the invention of the ECG in 1900, giving way to the recording of AF with ECG in the following decade [11]. The recognition of paroxysmal AF (pAF, terminates spontaneously or with intervention within 7 days of onset) and introduction of long-term anticoagulation for ischemic stroke prevention made it necessary to monitor patients longitudinally. The early outpatient external devices included Holter ECG monitors that could be carried for up to 72 h. Norman J. Holter, the “father” of all ambulatory monitoring, was the first to develop an unwieldy telemetry device for long-period, continuous recording of cardiac electric potentials in 1947 [12]. A truly portable, self-contained recorder was developed and marketed in 1963.

Over the past decade, longer-term continuous external monitoring devices such as mobile cardiac outpatient telemetry (MCOT) and cardiac patches became available and they were shown to increase the yield of AF detection. MCOT can send the data directly to a central monitoring station instead of recording it. The advantages of these devices are their external portable nature and ease of plugging/unplugging that allows their use for up to 1 month. Disadvantages include user (patient) dependence, sensitivity, issues with regular streaming as well as problems with reviewing/reporting. The patch monitors are devices designed without the wires or connecting electrodes to the recorder. They provide long-term monitoring of 14 days or longer with reasonably good patient adherence but with a limited localization ability and inconsistent optimal ECG signal quality because of closely spaced electrodes and varying body types. Unlike the implantable loop monitors, patients who receive MCOT or patch monitors are not necessarily followed by a cardiac electrophysiologist so the quality of reporting of a positive or negative study may not be optimal. The ordering physician not uncommonly receives alarms such as cardiac pauses or brief ventricular tachyarrhythmias, issues that are difficult to manage for the neurologist who does not primarily treat these cardiac conditions.

The other type of ambulatory ECG monitors are intermittent long-term loop recorders (1 to 36 months) which store the heart’s electrical signals only when the monitor is triggered by a patient or by abnormal heart rhythm. These loop recorders can be either external, worn around the waist or wrist, or implantable, inserted under the skin in the left parasternal region. Two multicenter RCTs (the EMBRACE trial and the CRYSTAL-AF trial) showed improvement in the detection of AF after ischemic stroke and TIA of undetermined cause with the use of external and internal loop recorders over 24-h Holter ECG, respectively [13, 14]. In the EMBRACE trial, AF lasting 30 s or longer was detected during 30 days of monitoring in 45 out of 280 patients (16.1%) with the use of an external loop recorder (ELR), against a detection rate of nine out of 277 (3.2%) in the control group (*P* < 0.001) [13].

In CRYSTAL-AF trial, 441 patients with a diagnosis of cryptogenic stroke or TIA of undetermined cause were randomly assigned to an internal loop recorder (ILR) (Fig. 1a) or a conventional ECG monitoring strategy. The study population had to have no evidence of AF during at least 24-h ECG monitoring before randomization within 90 days after the index event. The primary end point was the time to first detection of AF lasting 30 s or longer at 6 months. The time to first detection of AF within 12 months was one of the secondary end points. By 6 months, AF was detected in 19 patients (8.9%) randomized to the ILR group whereas in 3 patients (1.4%) in the control group (hazard ratio [HR], 6.4; 95% confidence interval [CI], 1.9 to 21.7; *P* < 0.001). By 12 months, AF was diagnosed in 12.4% of the patients in the ILR group (29 patients) versus 2.0% of the patients in the control group (4 patients) (HR, 7.3; 95% CI, 2.6 to 20.8; *P* < 0.001) [14]. The median time from randomization to detection of AF was 84 days in the ILR group and 53 days in the control group during the 12-month study period. Inspite of only 48 patients followed for 36 months, the rate of detection of AF was 8.8 times higher in the ILR group than the control group at 3-years follow-up. Overall, the results of CRYSTAL-AF demonstrate the importance of prolonged cardiac monitoring in many patients beyond the 30-day to detect AF in cryptogenic stroke patients. For long-term monitoring, ILRs are superior to all other approaches and their placement consists of a non-invasive outpatient procedure that can also be performed by neurologists. A recent randomized study showed 100% success during office (*n* = 251) or hospital (*n* = 231) insertions and very low complication rates, 0.8 vs 0.9% respectively [15•]. As the recent data established that long-term monitoring increase the yield of AF detection by many folds in high-risk populations, the relevance of AF duration and AF load has drawn interest in the field of neurology as well.

## The Duration of AF, AF Load, and Their Impact on Treatment Decisions

The longer-term AF monitoring systems resulted in the relatively common detection of short AF episodes. This advance challenged the previous paradigm that AF should last longer than 24 h to result in embolism formation [16]. Even though the duration of AF should be > 30 s for the current operational definition of AF recurrence [17], the shortest duration of AF that would predict embolization and therefore requiring antiembolic measures is currently debated. In recent years, atrial tachyarrhythmias lasting for at least 6 min in the absence of clinically diagnosed AF, termed as atrial high-rate episodes (AHREs), are increasingly recognized in patients presenting with stroke and TIA. In an observational prospective study investigating the prevalence of AHREs in 2580 patients with a recently implanted cardioverter defibrillator (ICD) device, 10.1% of the patients had AHREs and the presence of AHREs was predictive of stroke or systemic embolism even after adjustment for predictors of stroke (HR, 2.50; 95% CI 1.28–4.89) [18]. Daily AF load might also be an important factor to determine stroke risk, as atrial tachycardia/AF burden greater than 5.5 h on any given day conferred highest risk for embolic events (HR, 2.20; 95% CI 0.96–5.05) compared to no atrial tachycardia/AF [19]. In a pooled analysis of 10,016 patients with ICD devices [20], although all cutoff points of AF burden (5 min, 1, 6, 12, and 23 h) were associated with ischemic stroke, the highest risk was observed at the cutoff point of ≥ 1 to < 6 h (HR, 2.11, 95% CI 1.22–3.64). Ongoing studies will provide more data as to the optimal duration cutoff for a paroxysm of AF or even more broadly the AF load that would require the use of embolic prevention strategies in patients without prior ischemic stroke or TIA. Currently, the bulk of the data suggest using optimal stroke prevention measures in patients with even a brief AF episode if their embolic risk is moderate to high. In the classical neurological patient who had an ischemic stroke or TIA of a probable embolic source, the detection of AF of any duration should trigger a discussion of the best stroke prevention measure, i.e., OAC or LAAC.

## Determining the Embolic Risk in AF

Risk-stratification schemes are frequently used in patients with AF to predict the risk of embolic stroke. Of these, the CHADS_2_ (congestive heart failure, hypertension, age ≥ 75, diabetes, and prior stroke (doubled) and CHA_2_DS_2_-VASc [congestive heart failure, hypertension, age ≥ 75 years (doubled), diabetes mellitus, prior stroke or TIA or thromboembolism (doubled), vascular disease, age 65 to74 years, sex (female) category] scoring systems are used commonly in clinical and research practice. Although patients with the lowest risk may not be well identified with the use of these scoring systems, current guidelines recommend the use of CHA_2_DS_2_-VASc for the identification of “truly low-risk” AF patients who have low annual stroke rates of ≤ 1% [21, 22]. The ATRIA score is a recently proposed scoring system based on the most modern database among others, obtained from the Kaiser Permanente Northern California [23]. The risk factors in the ATRIA score are age (categorized as < 65, 65–74, 75–84, and ≥ 85 years), female sex, diabetes mellitus, heart failure, hypertension, proteinuria, and renal disease. A stroke history is not scored alone in this scheme, but higher scores are assigned in the increasing range of age groups among those with prior stroke. In spite of being a more complicated scoring system, which can be overcome with the use of computer or smart device applications, the ATRIA score predicts ischemic stroke risk better than CHADS_2_ or CHA_2_DS_2_-VASc, also with an enhanced ability for severe stroke prediction. A number of imaging findings such as the echocardiographic characteristics of the LAA and its blood flow can also help determining the embolic risk. Despite their shortcomings, there is a rationale to use the embolic risk scores in general. The great majority of symptomatic embolic events were ischemic strokes (about 90%) in validation studies, so this line of research is less likely to have a highly heterogenous end point such as the case with OAC-related hemorrhagic events. Based on the current guidelines, we use CHA_2_DS_2_-VASc but also review the ATRIA score in making management decisions in patients with NVAF. In more complicated situations where the choice of stroke prevention measure is not straightforward, imaging and other features can also be considered.

## Determining Hemorrhagic Risk and Its Relevance in AF Management

One very important issue to remember is that human beings are not embolic or hemorrhagic risk scores. Some of the shortcomings of embolic risk scores are discussed above but they got to be used as a general guide to understand ischemic risk for both OAC and LAAC decisions. Hemorrhages resulting from the use of life-long OAC treatment are a lot more heterogenous in terms of site, severity, and outcomes, to the point that “hemorrhagic risk scores” such as HAS BLED or HEMORR_2_HAGES are much less helpful. The physician caring for AF patient should always ask about any history of prior hemorrhage. If present, the site of the bleed should be clarified. It is imperative to contact the appropriate specialist for gastrointestinal, ocular, intra-articular, or other non-neurologic bleeds. The cause of such bleeds and whether the etiology was identified/treated, the recurrence risk and the feasibility of short and long-term anticoagulation in the individual patient are issues that need to be discussed with the appropriate specialist and documented in the chart. Similarly, the presence of hypocoagulable states, liver or renal failure or other systemic factors predisposing to hemorrhagic tendency should also be addressed with the appropriate disciplines.

Intracranial hemorrhage is by far the most feared complication of long-term systemic anticoagulation and the main reason for undertreatment of AF patients worldwide. One major recent advance was the FDA approval of LAAC using the WATCHMAN device in NVAF, a purely endovascular approach that circumvents the need for long-term anticoagulation in patients at higher than usual hemorrhagic risk. The neurologist should be well aware of the conditions associated with higher than usual ICH risk in order to have a meaningful shared decision-making discussion that includes LAAC, OAC, or other approaches with such AF patients.

OAC-ICH is a devastating condition associated with a high risk of in-hospital mortality (mortality 52% for OAC-ICH vs 25.8% for other ICHs) and poor outcomes, and accounts for nearly 25% of all ICHs [24]. Observational studies report that about 37% of the ICH patients have AF requiring anticoagulation for thromboembolic prevention [25]. About 70% of the OAC-ICH is due to the rupture of arteries/arterioles weakened by chronic cerebral small vessel disease (SVD) whereas most of the remainder are subdural hemorrhages (SDH) [26]. The location and presence of ICH (lobar versus deep) and associated neuroimaging markers, such as cerebral microbleeds (CMB) and cortical superficial siderosis (cSS), can help the clinicians to identify the dominant SVD type (Fig. 2) [27]. Patients with deep ICH and strictly deep-CMB are more likely to harbor hypertensive (HTN) SVD and their ICH recurrence risk is a non-trivial 2% annually (Fig. 2b) [27]. Patients 55 years or older with lobar ICH and one or more strictly lobar CMBs or cSS can be diagnosed with probable cerebral amyloid angiopathy (CAA) with high certainty, as long as alternative pathologies are ruled out (Fig. 2a, c, d) [28]. The presence of a single lobar ICH without any other hemorrhagic lesion in this context corresponds to a diagnosis of possible CAA per modified Boston criteria [29]. On average, CAA-ICH is associated with a 10% annual recurrence risk [28]. The presence and multifocality of cSS is associated with an incrementally higher ICH recurrence risk in CAA, up to 26.9% annually for multifocal or widespread cSS (Fig. 2d) [30]. About 20% of the primary ICH patients who receive a brain magnetic resonance imaging (MRI) have the concomitant presence of hematoma and CMBs in both deep and lobar areas (mixed-ICH), and their annual ICH recurrence risk was 5.1% [28].

Furthermore, CAA patients with strictly lobar-CMB without ICH show an important incidence rate of future ICH (5 per 100 person-years). In such patients, warfarin use has been shown to be an independent predictor of first ICH independently of other conventional risk factors [31]. As a separate cohort, about 25% of the patients who receive a brain MRI after an acute ischemic stroke or TIA are found to have microbleeds on GRE/SWI sequences. The future risk of ICH was 6-folds higher in those with microbleeds when compared to patients without, and such risk increased to 14-folds when 5 or more cerebral microbleeds were present [32]. The presence of severe white matter disease visible on T2/FLAIR sequences is also a risk factor for higher SVD-related ICH risk (Fig. 2a) [33]. A meta-analysis that included over 34,000 patients who had chronic/spontaneous SDHs showed an 11% recurrence risk during the first 2 years [34]. OAC use is a very significant risk factor for chronic/spontaneous SDH, so treatment approaches that do not require life-long anticoagulation should be considered in NVAF patients with past history of chronic SDH [35].

Overall, the identification of HTN-SVD, CAA, and related hemorrhage-prone markers such as CMBs, cSS, and severe white matter hyperintensities is clinically very important for the hemorrhagic risk stratification in AF patients. Any patient who gets an MRI should receive a hemosiderin sensitive sequence, called T2*/GRE/SWI in different MRI systems, and the neurologist should review these sequences to detect or rule out the presence of the hemorrhage-prone pathologies reviewed in this section. This effort is very important to determine the best stroke prevention option (OAC, LAAC, or others) for the individual AF patient, together with the embolic risk assessment.

## Pharmacological Treatment of AF

Warfarin, a vitamin K antagonist, has been used for stroke prevention in AF since the 1950s. A meta-analysis of RCTs showed that adjusted-dose warfarin reduced stroke risk by 64% compared to placebo while antiplatelet agents decreased it by 22% in AF patients [36]. For secondary prevention, an observational study of patients admitted after an acute ischemic stroke found a 27% recurrent stroke risk in patients with AF who did not receive warfarin, when compared to 18% among those with AF who received warfarin and 17% for patients without AF. The age-adjusted hazard ratio for stroke recurrence for non-anticoagulated AF was 2.1, whereas the hazard ratio for recurrent severe stroke was 2.4 [37].

Despite its established role in stroke prevention in AF, warfarin use presents a number of challenges such as the need for frequent blood draws to keep the international normalized ratio (INR) within the therapeutic range (lower values increase embolic whereas higher values the hemorrhagic risk), multiple drug and food interactions, and most importantly an increased risk of severe ICH. These problems prompted the search for safer anticoagulants and a direct thrombin inhibitor (dabigatran) as well as three activated factor X inhibitors (rivaroxaban, apixaban, edoxaban) proved to have clear advantages compared to warfarin in NVAF. They were non-inferior to warfarin for overall stroke prevention. They decreased ICH risk in study populations without past history of ICH or high hemorrhagic risk. They were easier to use, without the need for blood draws, and they had a lower risk of drug and food interactions [38–42]. All these DOACs are FDA approved, and they are increasingly used for AF-related stroke prevention in the USA and worldwide. A meta-analysis of RE-LY (dabigatran versus warfarin), ROCKET-AF (rivaroxaban versus warfarin), ARISTOTLE (apixaban versus warfarin), and ENGAGE AF–TIMI 48 (edoxaban versus warfarin) trials in NVAF revealed a 19% stroke risk reduction versus warfarin, mainly driven by a reduction in hemorrhagic stroke by 50% [38]. DOACs also reduced all-cause mortality, but dabigatran and rivaroxaban increased gastrointestinal bleeding. The disadvantages of DOACs are increased risk of gastrointestinal side effects and hemorrhage, increased bleeding risk especially in renal failure, availability and efficacy of specific antidotes, higher cost, and finally poor patient compliance [see Table 1].

**Table 1:** The efficacy, safety, and special considerations for direct oral anticoagulants (DOAC) and left atrial appendage closure (LAAC)

Study (DOAC)	Dosing	Mean age (+/− SD)/median (IQR)	Mean follow-up (years)	Mean CHADS_2_ (+/− SD)	Ischemic stroke, /100 patient-years HR vs. warf (95% CI)	All-cause mortality /100 patient-years HR vs. warf (95% CI)	Intracranial hemorrhage /100 patient-years HR vs. warf (95% CI)	Pros	Cons
RE-LY (dabigatran) * n* = 18,113	150 mg b.i.d	71.5 ± 8.8	2	2.1 ± 1.1	0.92 vs. 1.2% RR 0.76 (0.60–0.98) *p* = 0.03	3.64 vs. 4.13% RR 0.88 (0.77–1.00) *p* = 0.051	0.30 vs. 0.74% RR 0.40 (0.27–0.60) *P* < 0.001	- Reversal agent available	- GI side effects and GI bleeding risk - MI risk - Compliance
ROCKET-AF (rivaroxaban) * n* = 14,264	20 mg/day	73 (65–78)	1.94	3.5 ± 1	1.34 vs. 1.42% HR 0.94 (0.75–1.17) *p* < 0.581	1.9 vs. 2.2% HR 0.85 (0.70–1.02) *p* = 0.07	0.5 vs. 0.7%, HR 0.67 (0.47–0.93), *p* = 0.02	- Once daily dosing - Best studied DOAC in a high ischemic risk NVAF population	- GI bleeding risk - No reversal agent - Compliance
ARISTOTLE (apixaban) * n* = 18,201	5 mg b.i.d	70 (63–76)	1.8	2.1 ± 1.1	0.97 vs. 1.05% HR 0.92 (0.74–1.13) *p* = 0.42	3.52 vs. 3.94% HR 0.89 (0.8–0.998) *p* = 0.047	0.33 vs. 0.80% HR 0.42 (0.30–0.58), *p* < 0.001	- Better safety/efficacy profile among DOACs	- No reversal agent - Compliance
ENGAGE-AF (edoxaban) * n* = 21,105	60 mg/day	72 (64–78)	2.8	2.8 ± 1.0	1.25 vs. 1.25% HR 1 (0.83–1.19) *p* = 0.97	3.99 vs 4.35% HR 0.92 (0.83–1.01) *P* = 0.08	0.39 vs. 0.85% HR 0.47 (0.34–0.63), *P* < 0.001	- Once daily dosing	- Higher stroke risk if creatinine clearance > 95 mL/min - No reversal agent - Compliance
Study (LAAC)		Mean age (+/− SD)	Mean follow-up (years)	Mean CHADS_2_ (+/− SD)	Ischemic Stroke, /100 patient-years	CV/unexplained death /100 patient-years	Hemorrhagic stroke /100 patient-years	Pros	Cons
PROTECT AF (WATCHMAN) * n* = 707		72 ± 8.9	5	2.2 ± 1.2	1.35 vs 1.07% *p* = 0.49	1.03 vs. 2.32% *p* = 0.009	0.16 vs. 1.06% *p* = 0.005	- Obviates the need for life-long anticoagulation	- Requires an endovascular intervention with associated procedural risks
PREVAIL (WATCHMAN) * n* = 407		74.3 ± 7.4	5	2.6 ± 1.0	1.68 vs 0.73% *p* = 0.13	1.79 vs. 1.98% *p* = 0.76	0.18 vs. 0.54% *p* = 0.23		
Hazard ratios in the 5-year patient-level meta-analysis of the combined PROTECT AF and PREVAIL data					IS/SE – HR vs. warf (95%CI) 1.71 (0.94–3.11)	CV/unexplained death HR vs. warf (95%CI) 0.59 (0.37–0.94)	Hemorrhagic stroke HR vs. warf (95% CI) 0.20 (0.07–0.56)		

*HR* hazard ratio, *RR* relative risk, *CI* confidence interval, *SD* standard deviation, *IS* ischemic stroke, *SE* systemic embolism, *CV* cardiovascular

It is important to remember that current AF management guidelines recommend choosing the antithrombotic therapy based on a shared decision-making encounter after discussion of the absolute and relative risks of stroke and bleeding with the patient [1]. The CHA_2_DS_2_-VASc score is recommended for the assessment of ischemic stroke risk irrespective of the duration of NVAF or presence of atrial flutter [43]. Warfarin with target INR of either 2–3 or 2.5–3.5 is recommended for patients with AF, with the higher values for valvular AF. For NVAF and previous ischemic stroke or CHA_2_DS_2_-VASc score of two or greater, the initiation of warfarin or a DOAC is recommended [1]. Detailed suggestions are provided for reduced renal function, such as avoidance of DOACs and use of warfarin for patients on hemodialysis or with low creatinine clearance (< 15 mL/min).

However, real-world data shows that OACs are significantly underutilized globally for NVAF despite their overall benefit for stroke prevention and the increasing experience even with the novel agents [44, 45]. The fear of hemorrhagic complications and the commonly fatal OAC-ICH in particular is one of the most common reasons for such underutilization, in addition to the non-trivial shortcomings of both warfarin and DOACs discussed previously. An analysis of not only the embolic/ischemic but also the hemorrhagic risk (ICH risk in particular) should be performed for every patient with AF. Individualized stroke prevention decisions should be made based on a detailed discussion of benefits and competing risks with the patient and their family.

Intracranial hemorrhage is the most feared side effect of OAC, being related to very high rates of mortality and disability [46]. In a meta-analysis of 16 RCTs and 31 observational studies in patients receiving warfarin, the overall incidence of major bleeding was 2.1 per 100 patient-years (range, 0.9–3.4 per 100 patient-years) for RCTs and 2.0 per 100 patient-years (range, 0.2–7.6 per 100 patient-years) for observational studies [47]. Both RCTs and most of the real-world observational studies demonstrate a lower risk of ICH with the use of DOACs compared to warfarin [48]. However, it should be noted that no patient with a past history of ICH was included in the trials evaluating the efficacy and safety of DOACs. The lower overall ICH risk noted in the more recent studies seems to be related to better management of anticoagulation as well as risk factors such as hypertension and better detection and exclusion of patients at high ICH risk from the trials [49].

Various factors such as older age, labile INR, renal or liver dysfunction, alcohol consumption, concomitantly used drugs, and previous bleeding history increase the general risk of bleeding in patients treated with OAC. Of these, clinicians should especially focus on the modifiable risk factors to manage them more aggressively and to plan a closer follow-up. Stratification of the baseline ICH risk and how it is modulated with OACs is discussed in detail in the section above. These issues are particularly important for the neurologist to know, in order to determine whether life-long OAC or a non-pharmacological approach would be the most optimal stroke prevention measure for the individual patient.

## Left Atrial Appendage Closure

Because AF disrupts blood flow in LAA and over 90% of the atrial thrombi occur within this appendage, the concept of LAA closure (LAAC) emerged as a non-pharmacological stroke prevention approach in patients with NVAF. Historically; amputation of the LAA with the aim of thromboembolism prophylaxis was first suggested in 1952 in patients with mitral stenosis [50]. The excision or ligation of the LAA has been performed frequently for years during cardiac surgery, but the occlusion of the LAA percutaneously with a variety of devices has developed more recently. Of these, the WATCHMAN device (Boston Scientific, Natick, Massachusetts) has been tested in clinical trials against warfarin and is currently approved by FDA for stroke prevention in NVAF [Table 1]. LAAC and other current non-pharmacological approaches to stroke prevention in patients at high ICH risk have recently been extensively reviewed [51••]. The PROTECT AF (Watchman Left Atrial Appendage System for Embolic Protection in Patients With Atrial Fibrillation) was the first randomized trial comparing device-based LAAC to warfarin in patients with NVAF [52]. In this trial, the primary efficacy event rate was 3.0 per 100 patient-years (95% CI 1.9–4.5) in the intervention group and 4.9 per 100 patient-years (95% CI: 2.8–7.1) in the control group (RR:0.62, 95% CI 0.35–1.25), revealing the non-inferiority of LAAC to warfarin for the prevention of stroke, systemic embolism, or cardiovascular or unexplained death. In 3.8 years follow-up, the primary event rate was 2.3 events per 100 patient-years for WATCHMAN LAAC, compared with 3.8 events per 100 patient-years with warfarin (RR, 0.60; 95% CI, 0.41–1.05) [53]. These results met the criteria for both non-inferiority and superiority, compared with warfarin, for preventing the combined outcome of stroke, systemic embolism, and cardiovascular death, as well as superiority for cardiovascular and all-cause mortality. The subsequent PREVAIL (Watchman LAA Closure Device in Patients With Atrial Fibrillation Versus Long Term Warfarin Therapy) trial, including a higher risk group, also revealed the non-inferiority of LAAC compared to warfarin for late ischemic events (> 7 days after the procedure) [54]. Although non-inferiority was not achieved for overall efficacy in the PREVAIL trial essentially due to an overperforming warfarin arm (only 0.3 per 100-patient-years ischemic stroke rate during the initial 18 months follow-up), event rates were low and numerically comparable in both groups. This 0.3% ischemic stroke rate is far superior to that observed in any warfarin group of any clinical trial. The rates of ischemic stroke in the contemporary DOAC studies were 1.2 per 100 patient-years in RELY (Dabigatran), 1.42 in ROCKET AF (Rivaroxaban), 1.05 in ARISTOTLE (Apixaban), and 1.25 in ENGAGE-AF (Edoxaban). The WATCHMAN device was approved by the FDA in 2015. A recent meta-analysis of 5-years follow-up data from PROTECT AF and PREVAIL studies showed ongoing significant benefits in terms of lower hemorrhagic stroke, disabling/fatal stroke, cardiovascular/unexplained death, all-cause death, and post-procedure bleeding rates favoring LAAC [54]. The warfarin arm of PREVAIL continues to have unusually low ischemic stroke occurrences, but there was nevertheless no statistically significant difference in stroke and systemic embolism rates and finally the composite end point was similar comparing WATCHMAN to warfarin (hazard ratio 0.820; *p* = 0.27). Real-world post-approval experience shows high rates of successful WATCHMAN implantation with lower complication risks (pericardial effusion/tamponade, device migration). The incidence of procedure-related stroke or death was 0.08% each [55••, 56]. The benefit of stopping OAC about 6 weeks after the procedure and the abovementioned risks should be discussed with NVAF patients who are at high risk for ICH or other types of bleeding [55••, 56]. Dual antiplatelets are used for another 4.5 months, after which time the patients who had successful LAAC are kept on aspirin indefinitely. Aspirin was not related to high ICH risk in very large community based studies [57] and also in most patient cohorts at higher baseline ICH risk [30, 31]. It is also important to note that aspirin or other antiplatelets but not oral anticoagulation are the standard of care for most non-AF ischemic stroke etiologies (atherosclerosis of the cervical and intracranial vessels, cerebral small vessel diseases) and other common atherosclerotic cardiovascular conditions.

The effectiveness of prophylactic LAA exclusion, by sutures, staplers, or amputation, during cardiac surgery for reducing the risk of stroke has been evaluated in some studies, but with conflicting results [58]. More recently, an epicardial clip, AtriClip (AtriCure, Inc., Mason, OH), has become the preferred approach with high successful occlusion rates and no device-related complications at both short-term and long-term follow-up [59, 60]. This approach does not leave any device inside the heart but indeed requires a more invasive surgical procedure. Recent work suggests a clinical stroke prevention benefit from surgical LAAC when performed concomitantly with open-chest cardiac procedures [61]. Overall, the surgical LAAC methods may offer an anticoagulant sparing approach for select NVAF patients undergoing cardiac surgery for a different reason.

## Brief Updates on Valvular AF and Other Concomitant Pathologies

“Valvular AF” refers to patients with rheumatic valvular disease (predominantly mitral stenosis), a mechanical or bioprosthetic hearvalve. Stroke incidence rises up to 17-folds in AF patients in the presence of mitral valve disease [62]. In patients with mechanical heart valves (with or without AF) or AF related to rheumatic valve disease, warfarin is currently the only FDA-approved stroke prevention option [1, 63•]. When such patients have high ICH risk, medical management methods to decrease such hemorrhagic risk are used, including strict blood pressure control and avoidance of other offending drugs.

Atrial fibrillation is also common around the time of valvular, other cardiac or non-cardiac surgical procedures [64, 65]. Post-operative AF has been shown to be associated with the risk of stroke in most studies [66]. The decision to anticogulate in this setting and its duration should be decided in conjunction with the surgeon and cardiologist who were involved with the surgical procedure.

In patients with AF undergoing percutaneous coronary stenting (PCI), the administration of either low-dose rivaroxaban plus a P2Y12 inhibitor for 12 months or very low dose rivaroxaban plus dual antiplatelet therapy (DAPT) for 1, 6, or 12 months showed a lower rate of clinically significant bleeding when compared to standard therapy with a vitamin K antagonist plus DAPT for 1, 6, or 12 months [67••]. The three groups had similar efficacy rates, although the confidence intervals were broad, decreasing the strength of any conclusions regarding efficacy. Another recent study showed lower major or clinically relevant non-major bleeding in AF patients who underwent PCI who used dabigatran and a P2Y_12_ inhibitor (clopidogrel or ticagrelor) when compared to the triple antithrombotic therapy (warfarin, aspirin, and a P2Y_12_ inhibitor) over a mean 14 months follow-up. Dual therapy with dabigatran was non-inferior to warfarin triple therapy with respect to the risk of thromboembolic events [68••].

## Conclusions

Advances in the detection/management of AF and ICH risk stratification put the neurologist at the center stage for prevention of both ischemic and hemorrhagic strokes. New long-term outpatient cardiac rhythm monitoring systems including ILRs improved the sensitivity to detect AF for up to 3 years, enabling better informed decision-making. If AF is not present, the majority of the other stroke etiologies (atherosclerosis, small vessel disease) benefit from antiplatelet but not from anticoagulation therapy. If NVAF is detected, the neurologist should ideally be able to determine both embolic risk and risk of ICH or other hemorrhages. In patients with CHA2DS2-VASc of ≥ 2 and no elevated hemorrhagic risk, long-term OAC use should be discussed and the ischemic/hemorrhagic risk assessment should be repeated over years. Modern LAAC procedures such as FDA-approved WATCHMAN should be discussed as an anticoagulant sparing stroke prevention approach in NVAF patients at high risk for ICH based on an individual patient’s clinical and neuroimaging characteristics. Finally, for patients with valvular AF or history of mechanical valve replacement, warfarin is currently the only FDA-approved option.
